# The use of e-health and m-health tools in health promotion and primary prevention among older adults: a systematic literature review

**DOI:** 10.1186/s12913-016-1522-3

**Published:** 2016-09-05

**Authors:** Ramon Kampmeijer, Milena Pavlova, Marzena Tambor, Stanisława Golinowska, Wim Groot

**Affiliations:** 1 Department of Health Services Research, CAPHRI, Maastricht University Medical Center, Faculty of Health, Medicine and Life Sciences, Maastricht University, PO Box 616, 6200 MD Maastricht, The Netherlands; 2 Faculty of Health Sciences, Department of Health Economics and Social Security, Institute of Public Health, Jagiellonian University Collegium Medicum, ul. Grzegórzecka 20, 31-531 Krakow, Poland; 3 Top Institute Evidence-Based Education Research (TIER), Maastricht University, PO Box 616, 6200 MD Maastricht, The Netherlands

**Keywords:** Older adults, e-health, m-health, Telemedicine, Health promotion, Prevention

## Abstract

**Background:**

The use of e-health and m-health technologies in health promotion and primary prevention among older people is largely unexplored. This study provides a systematic review of the evidence on the scope of the use of e-health and m-health tools in health promotion and primary prevention among older adults (age 50+).

**Methods:**

A systematic literature review was conducted in October 2015. The search for relevant publications was done in the search engine PubMed. The key inclusion criteria were: e-health and m-health tools used, participants’ age 50+ years, focus on health promotion and primary prevention, published in the past 10 years, in English, and full-paper can be obtained. The text of the publications was analyzed based on two themes: the characteristics of e-health and m-health tools and the determinants of the use of these tools by older adults. The quality of the studies reviewed was also assessed.

**Results:**

The initial search resulted in 656 publications. After we applied the inclusion and exclusion criteria, 45 publications were selected for the review. In the publications reviewed, various types of e-health/m-health tools were described, namely apps, websites, devices, video consults and webinars. Most of the publications (60 %) reported studies in the US. In 37 % of the publications, the study population was older adults in general, while the rest of the publications studied a specific group of older adults (e.g. women or those with overweight). The publications indicated various facilitators and barriers. The most commonly mentioned facilitator was the support for the use of the e-health/m-health tools that the older adults received.

**Conclusions:**

E-health and m-health tools are used by older adults in diverse health promotion programs, but also outside formal programs to monitor and improve their health. The latter is hardly studied. The successful use of e-health/m-health tools in health promotion programs for older adults greatly depends on the older adults’ motivation and support that older adults receive when using e-health and m-health tools.

**Electronic supplementary material:**

The online version of this article (doi:10.1186/s12913-016-1522-3) contains supplementary material, which is available to authorized users.

## Background

In the healthcare sector, e-health and m-health tools are increasingly being used. E-health and m-health can be any kind of electronic device or monitoring system that is applied by physicians in the healthcare practice or by individuals to monitor or improve their health status. E-health typically refers to on-line and off-line computer-based applications while m-health refers to applications for mobile phones [1, 2]. Such tools can be used to stimulate a positive health behavior change or assist persons to lead a healthier life style, or to support diagnosis and treatment of diseases.

E-health and m-health technologies are mostly used by younger people. The potential of this technology for older adults is generally recognized although the application of e-health and m-health tools in health promotion and primary prevention for older persons has been largely unexplored [2].

Positive changes in health-related lifestyle among older adults offer the opportunity for health benefits. It is recognized that promoting health among this population group may contribute to more healthy life years and increased life expectancy [3]. Many diseases among older adults are partly or fully preventable if individuals engage in a healthy lifestyle [4]. For example, physical activity and a proper diet can help to prevent obesity, heart diseases, hypertension, diabetes and even premature mortality [5, 6]. Although the importance of a healthy lifestyle is known, older adults (50+ years) are frequently physically inactive [3, 7]. The use of e-health and m-health technologies could help older adults to improve or maintain their health. But to what extent is the use of such tools by older adults reported in the literature?

This study aims to provide insight in the scope of the use of e-health and m-health tools for health promotion and primary prevention among older adults using the method of a systematic review. Hitherto, no in-depth overview of this topic is provided in the literature. Therefore, our review is an initial step that explores the scope of the use of e-health and m-health tools in health promotion and primary prevention among older adults. We include the use of such tools within health promotion programs as well as the use of such tools by older adults outside formal programs with the goal to monitor and improve their health. In this way, our review may provide a base for subsequent more specific reviews focused on a certain type of e-health and m-health tools, and its use in health promotion programs, as well as reviews on the effectiveness of e-health and m-health promotion programs within a specific group of older adults and specific setting.

Our review focuses on two dimensions: the characteristics of e-health and m-health tools used for monitoring and improving the health of older adults and the determinants of the use of e-health and m-health tools by older adults. The review identifies gaps in the research area that can be used for setting up new studies as mentioned above.

The review is relevant for policy and society because of the ageing of the population and the increase in multi-morbidity which consequently lead to greater demand for healthcare and higher healthcare expenditure. Therefore, it is important to know whether and how the emerging new technologies, specifically e-health and m-health tools, can be used by older adults to prevent diseases and help older adults to have not only longer but also a healthier life.

## Methods

This study uses a systematic literature review to analyze the use of e-health and m-health tools for health promotion among older adults. The methodology of a systematic literature review outlined in Grant and Booth (2009) is applied [8]. The study starts with a systematic literature search based on predefined search terms and selection criteria. Then, the articles selected for the review are appraised and their relevant findings are synthesized narratively based on the objective of the review, with the support of descriptive tables. The applied review method allows for a comprehensive overview of the current knowledge in a specific research field. This distinguishes our systematic review from other review methods such as scoping review and meta-analysis.

Given the aim for this review, three components are used to build the search terms for the identification of studies on the use of e-health and m-health tools in health promotion and primary prevention among older adults: (1) elderly or old or senior; (2) health promotion or primary prevention; (3) telemedicine or e-health or m-health. Different forms of the above words as well as relevant synonyms are taken into account. This results in the following chain of keywords, which is used to search for relevant literature in search engine PubMed:

*(“aged”[MeSH Terms] OR “aged”[All Fields] OR “elderly”[All Fields] OR “old”[All Fields] OR “senior”[All Fields] OR “seniors”[All Fields])****AND****(“health promotion”[MeSH Terms] OR “health promotion”[All Fields] OR “promotion”[All Fields] OR “primary prevention”[MeSH Terms] OR “primary prevention”[All Fields] OR “prevention”[All Fields])****AND****(“telemedicine”[MeSH Terms] OR “telemedicine”[All Fields] OR “tele-medicine”[All Fields] OR “telehealth”[All Fields] OR “tele-health”[All Fields] OR “mhealth”[All Fields] OR “m-health”[All Fields] OR “ehealth”[All Fields] OR “e-health”[All Fields])*

In the search, MeSH terms (bibliographic thesaurus) were also included to ensure uniformity and consistency in the literature search. The search was done in October 2015.

Various inclusion and exclusion criteria are applied. To be included in the review, the publication should be published in the last 10 years, should be in English, and the full-paper can be obtained. There are no limitations with regard to the institution, which provides the e-health or m-health tools, i.e. papers that present e-health and m-health tools provided by state, insurers, employers and others are considered as relevant. We include papers which present the application of e-health and m-health tools not only within health promotion programs but also the use of such tools by older adults outside formal programs with the goal to monitor and improve their health. The publications could present data collected among older adults or among healthcare providers who provide services to older persons. Publications that discuss the topic in general as well as opinion papers and editorials are excluded.

Also, publications are selected if the age of participants (study group) is 50 years or older and if the focus is on health promotion and primary prevention. Based on Kenkel (2000), health promotion and primary prevention are defined in this review as activities that aim to reduce the probability of illness by stimulating a healthy lifestyle and providing services that might decrease the future incident of illnesses [9]. Hence, publications that deal with the use of telemedicine in home care to assist disable persons are excluded, as well as publications that report on the use of electronic devices and computer-based systems in secondary and tertiary prevention (e.g. monitoring of chronic conditions in case of specific diseases).

The first screening of the publications that appear after searching in PubMed with the chain of keywords given above, is based on the title and abstract of the publications. At this stage, publications are considered potentially relevant if their title and abstract have a link with the review topic. For the second screening, the publications are downloaded and the text of the publication is fully screened. Publications that fit the inclusion criteria outlined above are classified as relevant and are selected for the review.

After the screening, the method of directed (relational) content analysis of Hsieh and Shannon (2005), is used for the analysis of the publications [10]. This type of analysis requires the identification of categories (themes) relevant to the review objective, extraction of information related to categories and synthesis of the information classified in each category. The groups of themes that are used for the review and which form the units of analysis, are:

- ▪ Characteristics of the e-health and m-health tools: types of e-health tools (what tools and for what?); application of e-health tools (what scheme/program/initiatives?); target group or user group (who use e-health and m-health tools?); characteristics of the setting (where and what location/country features?)
- ▪ Determinants of the use of e-health and m-health tools/programs: facilitating factors for the use (what motivates and enables the use?); barriers to the use (what prevents or discourages the use?)

Based on these groups of themes, the data extraction is done. The results are presented per group of themes in a narrative manner and are complemented by descriptive tables. The quality of the publications (research design and findings of the study reported) is assessed in a qualitative manner. We classify a study as reliable if the methods of data collection and analysis are well defined in the publication, and are potentially repeatable. Similarly, we classify a study as valid if the publication provides clear indications of consistency of the results with stated study hypotheses, expectations and/or results of other similar studies. The generalizability of the study is defined based on indications for possible extrapolation of the findings to the larger population. The quality of this review is also checked using the Prisma 2009 checklist (see Additional file 1).

## Results

**Fig. 1:**
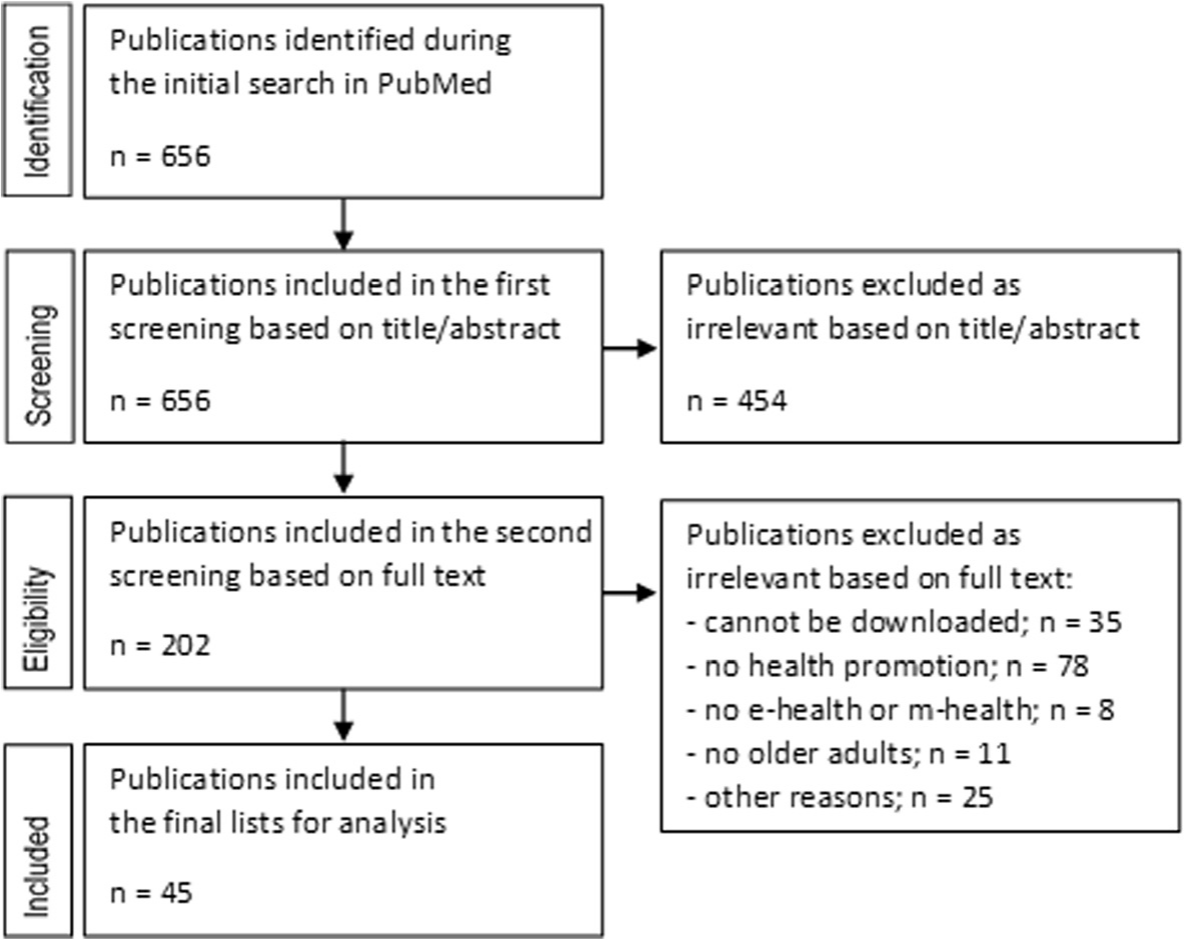
Search results and publication selection procedures

The chain of keywords shown above yields 656 publications, which are included in the initial screening. The results of the screening are presented in Fig. 1. In the first screening, 454 publications are excluded after reading the abstract based on the inclusion/exclusion criteria. In total, 202 publications are included in the second screening. For the second screening, the publications are downloaded. The full-paper cannot be obtained for 35 publications, and hence, these articles are excluded. The text of the remaining 167 articles is reviewed. From these 167 articles, 122 publications are excluded after reading the full text. The reasons of exclusion are: (1) publications are not about health promotion or primary prevention; (2) there is no e-health or m-health tool studied; (3) older adults are not a study group; (4) a combination of these above reasons. Thus, after the second screening, 45 publications are selected for this systematic review. A detailed description of the articles is presented in Additional file 2.

### General description of the selected publications

The main characteristics of the included publications are presented in Table 1. The majority of the publications have been published in the last 4 years. In the last 2 years only, 33.3 % of the publications are published. The majority of the studies have an explanatory aim (quantitative studies investigating relations and determinants) and only nine publications are explorative or descriptive (qualitative or mix-methods studies providing more insight on the topic).

**Table 1:** General description of publications included in the analysis (45 publications reviewed)

Classification category	Sub categories	N (%)	Reference index in Additional file 2
Year of publication	2014–2015	15 (33.3)	1, 2, 3, 4, 5, 6, 7, 8, 9, 10, 11, 12, 13, 20, 28
	2012–2013	12 (26.7)	14, 15,16,17, 18, 19, 21, 22, 23, 24, 25, 26,
	2010–2011	6 (13.3)	27, 29, 30, 31, 32, 33,
	2008–2009	6 (13.3)	34, 35, 36, 37, 38, 44
	2006–2007	6 (13.3)	39, 40, 41, 42, 43, 45
Aim/type of study	Descriptive	6 (13.3)	6, 8, 21, 23, 31, 38
	Explorative	3 (6.7)	1, 5, 27,
	Explanatory	36 (80)	3, 4, 6, 7, 9, 10, 11, 13, 14, 15, 16, 17, 18, 19, 20, 22, 24, 25, 26, 28, 29, 30, 32, 33, 34, 35, 36, 37, 39, 41, 40, 42, 43, 44, 45
Research approach	Qualitative (primary data)	6 (13)	2, 5, 7, 23, 31, 38
	Quantitative (primary data)	32 (69.6)	3, 9, 10, 11, 12, 14, 15, 16, 17, 18, 19, 20, 22, 24, 25, 26, 27, 28, 29, 32, 33, 34, 35, 36, 37, 39, 40, 41, 42, 43, 44, 45
	Mixed (primary data)	3 (6.5)	1, 4, 30
	Desk research (secondary data)	5 (10.9)	6, 8, 12, 13, 21
Design	Qualitative	7 (15.2)	1, 2, 5, 7, 23, 31, 38
	Quantitative - randomized controlled trial	28 (60.9)	3, 4, 9, 10, 11, 12, 14, 15, 16, 17, 18, 19, 20, 22, 24, 25, 26, 27, 28, 29, 33, 34, 37, 40, 41, 42, 43, 44
	Qualitative - not randomized controlled trial	9 (19.6)	6, 13, 23, 30, 32, 35, 36, 39, 45
	Systematic review	2 (4.3)	8, 21
Data collection/design	Observations	1 (2)	1
	Focus group discussion	2 (4)	2, 16
	Unstructured/semi structured interviews	7 (13))	1, 3, 5, 7, 31, 38, 40
	Standardized questionnaires/interviews/surveys	9 (17	11, 14, 18, 20, 30, 33, 37, 42, 43
	Online web based questionnaires/assessments	10 (19)	4, 10, 17, 25, 26, 27, 28, 29, 40, 44
	Secondary data/patient records	5 (10)	8, 9, 12, 13, 21
	Test results/self-report	18 (35)	6, 11, 15, 18, 19, 22, 23, 24, 25, 32, 33, 34, 35, 36, 37, 39, 41, 45
Reliability//	Reliability is clear	19 (42.2)	1, 2, 5, 7, 8, 12, 14, 15, 16, 20, 21, 22, 24, 27, 38, 40, 41, 43, 44
	Reliability is unclear	22 (48.9)	6, 9, 10, 11, 13, 17, 18, 19, 23, 25, 28, 29, 31, 32, 33, 34, 35, 36, 37, 39, 42, 45
	Reliability is not analyzed	4 (8.9)	3, 4, 26, 30,
Validity	Validity is clear	16 (35.6)	1, 2, 3, 5, 8, 15, 16, 20, 21, 30, 36, 38, 41, 42, 43, 44
	Validity is unclear	22 (48.9)	4, 6, 9, 11, 13, 14, 18, 19, 22, 23, 24, 25, 28, 29, 31, 32, 34, 35, 37, 39, 40, 45
	Validity is not analyzed	7 (15.6)	7, 10, 12, 17, 26, 27, 33
Generalizability	Generalizability is clear	11 (24.4)	1, 5, 6, 8, 9, 14, 16, 22, 42, 43, 45
	Generalizability is unclear	20 (44.4)	2, 3, 11, 12, 13,17, 18, 19, 20, 24, 25, 28, 29, 31, 34, 37, 39, 40, 41, 44
	Generalizability is not analyzed	14 (31.1)	4, 7 10, 15, 21, 23, 26, 27, 30, 32, 33, 35, 36, 38,

The sum of N per category can exceed 45 as papers can be classified in multiple sub-categories

There are four different research approaches in the publications reviewed. The majority (32 publications) are quantitative studies with primary data collection. Five publications use secondary data. In 28 publications, randomization was reported. There are two systematic reviews conducted, both are about telehealth. Seven methods of data collection are reported. Most publications use biomedical test results as input data. These test results are measured by a healthcare professional or are provided by the participant through a self-report. Five publications, including the two systematic reviews, use secondary data or patient records for data analysis. In one publication, observation techniques are used for the data collection. Focus group discussions are reported in two publications. Seven publications report unstructured or semi-structured interviews for data collection. The other publications use standardized questionnaires or online questionnaires.

In Table 1, our qualitative assessment of reliability, validity and generalizability is also presented. If the publications are clear about their methods of data collection and analysis, they are considered reliable. In total, 19 publications have a clear and reliable description of the methods. In 16 publications, some aspects of validity are mentioned. Generalization is clearly outlined in 11 publications.

### Characteristics of the use of e-health and m-health tools among older adults

In Table 2, the characteristics of the e-health and m-health tools used for health promotion and primary prevention among older adults are outlined. In the first category, the type of tool is mentioned. In 21 publications, a website is reported for e-health services. One website for instance, offers a help program to reduce weight, where participants can enter their data and plan their goals. Then, the website helps with feedback to achieve these goals. Other website-related interventions deliver information for health prevention or health promotion. Two publications report on a smartphone app. In 15 publications, the use of various devices is reported. These devices are often used to gather health-related data, for example, a pedometer to count steps. There are 4 publications that report on the use of video consults so that patients do not need to go to a healthcare facility. Participants can use programs like Skype to have a video consultation with the nurse or general practitioner. In 13 publications, the use of telehealth is reported. Telehealth is used to deliver online webinars. Here, people can participate in a course or program. There are webinars to help older adults to get active or to work on their healthy behavior.

**Table 2:** Characteristics of e-health and m-health tools used in health promotion and primary prevention among older adults

Classification category	Sub categories	N (%)	Reference index in Additional file 2
Type of e-health and m-health tools	Apps	2 (3.6)	1, 26
	Website	21 (38.2)	2, 5, 6, 9, 10, 14, 15, 16, 17, 19, 22, 25, 27, 28, 29, 35, 37, 38, 40, 43, 44
	Device	15 (27.3)	3, 4, 7, 11, 12, 15, 21, 22, 24, 31, 32, 33, 36, 37, 42
	Video consult (skype)	4 (7.3)	8, 39, 41, 45
	Webinars	13 (23.6)	3, 8, 13, 18, 20, 21, 23, 25, 28, 29, 30, 32, 34,
Type of use of e-health and m-health tools	Use without program (phone diary)	1 (2.2)	26
	Use in computer tailored lifestyle program	18 (40)	2, 6, 9, 12, 14, 15, 17, 18, 22, 24, 25, 27, 28, 29, 35, 37, 43, 44
	Use in program providing support/feedback (Internet/face-face)	10 (22.2)	1, 3, 4, 7, 10, 11, 16, 23, 34, 36
	Use in online health information	4 (8.9)	5, 19, 38, 40
	Use in telehealth programs	12 (26.7)	8, 13, 20, 21, 30, 31, 32, 33, 39, 41, 42, 45
Study group^a^	No specific requirement	17 (37)	2, 4, 7, 9, 16, 17, 20, 21, 30, 31, 32, 33, 36, 39, 40, 41, 44
	Women only	3 (6.5)	3, 10, 26
	With risk/signs of specific disease	4 (8.7)	1, 19, 35, 45
	Physical conditions (overweight)	14 (30.4)	6, 10, 11, 12, 13, 18, 22, 25, 27, 28, 29, 34, 37, 43
	(Risk) Behaviors-lifestyle	3 (6.5)	14, 23, 24
	Cultural group (migrants/Latino’s/African American)	3 (6.5)	5, 15, 42
	Based on setting: in clinics/community	1 (2.2)	8
	Low computer knowledge	1 (2.2)	38
Country location	Europe	10 (22.2)	1, 2, 7, 14, 19, 21, 26, 27, 32, 44
	United states	27 (60)	3, 4, 8, 9, 10, 11, 12, 13, 15, 16, 17, 20, 22, 23, 24, 25, 28, 29, 30, 31, 33, 34, 35, 40, 42, 43, 45
	Canada	3 (6.7)	6, 39, 41
	Australia	2 (4.4)	5, 18
	Asia	3 (6.7)	36, 37, 38

The sum of N per category can exceed 45 as papers can be classified in multiple sub-categories

^a^All groups are older adults (at least 50 years old)

Virtually all e-health and m-health tools reported are related to a health promotion or primary prevention program for older adults. Only, one publication describes a tool without a health promotion or prevention program, this tool is a phone-based diary (app). The majority of the publications report on computer tailored lifestyle programs (computer-based e-health programs). Specifically, a computer tailored lifestyle program has the aim to change unhealthy behaviors. Such program helps older adults with personal goal setting and achieving these goals. In addition, ten programs that we identified, are based on providing feedback. Feedback is provided with an interactive voice response, with the use of internet, or it is a face-to-face feedback. These programs do not have to be tailor-made but could provide the same feedback for an entire group. Telehealth offers different programs that are studied in 12 publications. There are telehealth programs to increase physical activity among older adults. Other telehealth programs provide information, for example, on stroke prevention. Telehealth programs help older adults to access their health. To increase information for health prevention or health promotion among older adults, 4 publications report on the use of online health information.

The third category in Table 2 portrays the study groups. All publications report on the use of e-health and m-health tools among older adults, older adults being defined as 50 years and older. The majority of the publications (17 publications) do not have further limitations of the study group. Fourteen publications mention specific physical requirements. Most of them are health programs that aim at people who suffer from overweight. There are three studies focused on older women, and three that aim at older adults from a specific cultural group. One publication reports on older adults with limited computer knowledge.

The majority of the publications come from the United States (27 out of the 45 publications reviewed). Ten publications come from Europe. There are three publications from Asia, of these, two are from Japan, and one from Hong Kong. The other five publications come from Canada and Australia.

In Table 3, a cross-tabulation is given to show which types of e-health and m-health tools in what programs are reported. Apps are used for the provision of health-related feedback to older adults within a health promotion program, as well as outside a formal program. Websites are also used within health promotion programs to provide health-related information to older adults. Websites, devices and webinars are used in computer tailored lifestyle programs and program providing health-related support or feedback. Telehealth programs involve the use of devices, video consults and webinars.

**Table 3:** Cross-tab for type of e-/m-health tools and type of use of e-/m-health tools among older adults

Type of e-health and m-health tool	Type of use of e-health and m-health tools	N (%)	Reference index in Additional file 2
Apps	Tool without program	1 (50)	26
	Computer tailored lifestyle program	-	-
	Program providing support/feedback	1 (50)	1
	Online health information	-	-
	Telehealth	-	-
Website	Tool without program	-	-
	Computer tailored lifestyle program	15 (71.4)	2, 6, 9, 14, 15, 17, 22, 25, 27, 28, 29, 35, 37, 43, 44
	Program providing support/feedback	2 (9.5)	10, 16
	Online health information	4 (19.1)	5, 19, 38, 40
	Telehealth	-	-
Device	Tool without program	-	-
	Computer tailored lifestyle program	5 (33.3)	12, 15, 22, 24, 37
	Program providing support/feedback	5 (33.3)	3, 4, 7, 11, 36
	Online health information	-	-
	Telehealth	5 (33.3)	21, 31, 32, 33, 42
Video consult (skype)	Tool without program	-	-
	Computer tailored lifestyle program	-	-
	Program providing support/feedback	-	-
	Online health information	-	-
	Telehealth	4 (100)	8, 39, 41, 45
Webinars	Tool without program	-	-
	Computer tailored lifestyle program	4 (30.8)	18, 25, 28, 29
	Program providing support/feedback	3 (23.1)	3, 23, 34
	Online health information	-	-
	Telehealth	6 (46.1)	8, 13, 20, 21, 30, 32

The sum of N per category can exceed 45 as papers can be classified in multiple sub-categories

In Table 4, a cross-tabulation is given to show which types of e-health and m-health tools are reported in which year and in which country. For Asia, there are three publications, each from the period 2008–2009. They all report on the use of e-health tools in programs. For Canada, there are two publications in the period 2006–2007, both about telehealth. The other publication in Canada is published in the period 2014–2015 and is about the use of web-site in a computer-tailored lifestyle program.

**Table 4:** Cross-tab for year, country and type of use of e-health and m-health tools among older adults

Country or location	Year of publication	Type of use of e-health and m-health tools	N (%)	Reference index in Additional file 2
Europe	2014–2015	Computer tailored lifestyle program and program providing support/feedback	3 (30)	1, 2, 7,
	2012–2013	Computer tailored lifestyle program, online health information, use without program	3 (30)	14, 19, 26
	2010–2011	Computer tailored lifestyle program and telehealth	2 (20)	21, 27
	2008–2009	Computer tailored lifestyle program and telehealth	2 (20)	32, 44
	2006–2007		-	-
United states	2014–2015	Computer tailored lifestyle program, telehealth, and program providing support/feedback	10 (37)	3, 4, 8, 9, 10, 11, 12, 13, 20, 28
	2012–2013	Computer tailored lifestyle program and program providing support/feedback	7 (25.9)	15, 16, 17, 22, 23, 24, 25
	2010–2011	Computer tailored lifestyle program and telehealth	4 (14.8)	29, 30, 31, 33,
	2008–2009	Computer tailored lifestyle program and program providing support/feedback	2 (7.4)	34, 35,
	2006–2007	Online health information, computer tailored lifestyle program and telehealth	4 (14.8)	40, 42, 43, 45
Canada	2014–2015	Computer tailored lifestyle program	1 (33.3)	6
	2012–2013		-	-
	2010–2011		-	-
	2008–2009		-	-
	2006–2007	Telehealth	2 (66.7)	39, 41
Australia	2014–2015	Online health information	1 (50)	5
	2012–2013	Computer tailored lifestyle program	1 (50)	18
	2010–2011		-	-
	2008–2009		-	-
	2006–2007		-	-
Asia	2014–2015		-	-
	2012–2013		-	-
	2010–2011		-	-
	2008–2009	Program and program providing support/feedback, computer tailored lifestyle program and online health information	3 (100)	36, 37, 38
	2006–2007		-	-

The sum of N per category can exceed 45 as papers can be classified in multiple sub-categories

The majority of the publications (17 publications) are published in the last 4 years and most come from the United States. The main focus in the US studies is on the use of e-health and m-health tools in computer-tailored lifestyle programs for older adults. In Europe, the number of publications focused on older adults that we reviewed did not increase much during the past 10 years. There is an increased focus on programs based on the use of e-health tools for providing support and feedback for a healthy lifestyle. From the 10 publications with such focus, eight have been published in the last 4 years. These publications are from the USA and Europe. E-health tools in telehealth programs are reported before 2012.

Table 5 presents a cross-tabulation of the study group and the type of use of e-health and m-health tools. Most publications with a specific study group are about physical conditions. There is only one publication that reports on telehealth that aims to help older adults who live in a clinic. The only publication that is focused on older adults with limited computer knowledge is a program about online health information. Some publications report on the use of e-health and m-health tools in computer-tailored lifestyle programs to help older adults get physically active. Other publications for older adults with specific physical conditions report on the use of e-health and m-health tools in programs focused on providing support and feedback, and one publication is focused on telehealth. Studies on telehealth most often include older adults in general. Three publications report on the use of e-health and m-health tools by older women in a computer-tailored lifestyle program and programs focused on providing health-related support and feedback.

**Table 5:** Cross-tab for study group and type of use of e-health and m-health tools among older adults

Study group^a^	Type of use of e-health and m-health tools	N (%)	Reference index in Additional file 2
No specific study group	Tool without program	-	-
	Computer tailored lifestyle program	5 (25)	2, 6, 9, 17, 44
	Program providing support/feedback	3 (15)	4, 7, 16
	Online health information	4 (20)	40
	Telehealth	8 (40)	20, 21, 30, 31, 32, 33, 39, 41
Women only	Tool without program	1 (33.3)	26
	Computer tailored lifestyle program	-	-
	Program providing support/feedback	2 (66.7)	3, 10
	Online health information	-	-
	Telehealth	-	-
With risk/indication of specific disease	Tool without program	-	-
	Computer tailored lifestyle program	1 (33.3)	35
	Program providing support/feedback	-	-
	Online health information	1 (33.3)	19
	Telehealth	1 (33.3)	45
Physical conditions (overweight)	Tool without program	-	-
	Computer tailored lifestyle program	10 (71.4)	6, 12, 18, 22, 25, 27, 28, 29, 37, 43
	Program providing support/feedback	3 (21.4)	10, 11, 34
	Online health information	-	-
	Telehealth	1 (7.1)	13
(Risk) Behaviors-lifestyle	Tool without program	-	-
	Computer tailored lifestyle program	2 (66.7)	14, 24
	Program providing support/feedback	1 (33.3)	23
	Online health information	-	-
	Telehealth	-	-
Specific cultural group	Tool without program	-	-
	Computer tailored lifestyle program	1 (33.3)	15
	Program providing support/feedback	-	-
	Online health information	1 (33.3)	5
	Telehealth	1 (33.3)	42
Based on setting: In clinics/community	Tool without program	-	-
	Computer tailored lifestyle program	-	-
	Program providing support/feedback	-	-
	Online health information	-	-
	Telehealth	1 (100)	8
Low computer knowledge	Tool without program	-	-
	Computer tailored lifestyle program	-	-
	Program providing support/feedback	-	-
	Online health information	1(100)	38
	Telehealth	-	-

The sum of N per category can exceed 45 as papers can be classified in multiple sub-categories. ^a^All groups are older adults (at least 50 years old)

### Facilitating factors and barriers to the use of e-health and m-health tools in health promotion among older adults

For the use of e-health and m-health tools, barriers and facilitating factors are reported in the publications reviewed. These factors are described in Table 6. It should be underlined however, that most of the factors listed in Table 6, such as motivation, self-regulation, information and rewards, are important determinants of behavior change in general and not necessarily direct determinants of the use of e-health and m-health tools per se. At the same time, other factors in Table 6, such as usability and accessibility can be directly related to the use of e-health and m-health tools.

**Table 6:** Factors influencing the use of e-health and m-health tools for health promotion and primary prevention among older adults

Classification category	Sub categories	N (%)	Reference index in Additional file 2
Facilitating factors	Motivation/support/feedback	12 (35.3)	1, 2, 3, 4, 12, 18, 21, 29, 30, 34, 39, 43
	Self-regulation/goal setting	4 (11.8)	9, 17, 18, 36
	Information (progress, usefulness, awareness)	2 (5.9)	1, 31
	Reward (financial, noticed physical change)	3 (8.8)	4, 6, 39
	Usability	3 (8.8)	1, 7, 26
	Accessibility (language, form – online or print)	2 (5.9)	5, 9
	Remote help at home (no travel distance)	8 (23.5)	13, 28, 29, 30, 31, 33, 35, 39
Barriers to use	Personal choice lack of time/priority/cost	6 (20)	2, 3, 17, 23, 24, 34
	Lack of adherence or motivation/support	8 (26.7)	3, 4, 12, 17, 24, 34, 42, 44
	Unclear device or information/wrong interpretation/lack of guidance	3 (10)	1, 7, 38
	Barriers related with technology/device	4 (13.3)	1, 3, 26, 45
	Socio demographical barriers (age, educational level, skills with electronic device)	6 (20)	2, 5, 15, 17, 38, 45
	Lack or resource for telecare	1 (3.3)	31
	Policy/reimbursement changes required	2 (6.7)	28, 29

The sum of N per category can exceed 45 as papers can be classified in multiple sub-categories

Seven types of facilitating factors are reported in the publications. The most often mentioned facilitating factors are motivation, support and feedback. These are reported in 12 publications. Specifically, support received from other participants in the e-health or m-health program is a key factor to help to change behavior. Motivation or feedback from other participants is also important to observe progress. This also contributes to adherence to the e-health or m-health programs. Four publications indicate that it is necessary to let older adults participate in accordance with their own planning to change. This could be accomplished by self-regulation and goal setting. Goal setting and insight in how they perform, is a way to keep them motivated. Information on individual progress and the nature of the tool also help to facilitate the use of the tool. According to three publications, it is helpful if there is a reward system. The reward system can be based on both the use of the e-health/m-health tool and concrete changes in health-related behavior. This could be a financial reward, or a reward in the sense that the participants can notice progress. Three publications mentioned user-friendliness as facilitating factors of the tools. If the electronic device is simple and works easily, older adults are more willing to keep using it. Two publications indicate the accessibility to the tools or programs as a facilitating factor. It is be better if the programs or tools are provided in multiple languages so that older adults could use it in their native language. For some older adults, it is better to have access to different forms of information, for example, if there is also a printed version beside the online information. Access to remote help at home is the second most often mentioned facilitating factor. This is the case in eight publications. The benefit of this remote help at home is often the lack of travel distance.

In the publications, barriers to the use of e-health and m-health tools for health promotion and primary prevention among older adults are also mentioned. These barriers are presented in Table 6. There are seven categories of barriers. The first two categories are related to personal barriers. Six publications mention barriers to use of e-health and m-health tools related to personal choice. This choice refers to the lack of time or other priorities. A solution that is indicated is to have a tool that can be paused and the use can be resumed when the older adult has time. Some publications mention that the monetary costs of use are too high. As mentioned for the facilitating factors, the lack of motivation and support is also most often reported as a barrier to the adherence to e-health or m-health health promotion programs. When there is an online support group, the group could be used to motivate each other. If the online support group is not used or only filled with negative comments, then the support has a negative influence and becomes a barrier. According to the publications reviewed, there is also a lack of motivation when participants cannot reach their goals. When devices are used or information is provided, it should be clear how the device works and the users should be able to understand the information that they receive. The lack of information or the lack of comprehensible information is mentioned in three publications. Barriers related to the technology used and the device is reported in four publications. There are examples of problems with the use of internet or with the device. Problems with the electronic devices can also be caused by sociodemographic barriers. Sometimes older adults do not have the proper skills to work with e-health or m-health devices. In four publications, sociodemographic barriers are mentioned. The sociodemographic barriers are related to educational level and age. Three publications indicate barriers that are related to policy or to a lack of resources to implement the e-health program or tool.

## Discussion

This systematic literature review presents evidence on the scope of the use of e-health and m-health tools for health promotion and primary prevention among older adults, as well as the factors that influence the use of these tools. There are different kinds of e-health and m-health tools used for health promotion and primary prevention among older adults. These include apps, websites, devices, video consults and webinars. Many of the health promotion and primary prevention programs for older adults that utilize such tools, have websites with information on health-related aspects. This is for example the case with computer-tailored lifestyle programs and telehealth programs. The majority of the publications on e-health and m-health tools that we reviewed, study the general older adult population. Only few publications report studies focused on a specific older adult group. The most common specific study group consists of older adults with a certain physical limitation, most often the need of weight reduction. This is not surprising as many diseases can be prevented through physical activity or maintenance of a healthy weight [11–13]. This could explain why there is a strong focus on older adults with weight problems as a study group. The publications with this study groups most often report the use of e-health and m-health programs in a computer tailored lifestyle program. This is also reported by the systematic reviews focused on e-health interventions for physical activity and dietary behavior change [14].

During the past 10 years, the amount of publications reporting on the use of e-health and m-health tools in health promotion and primary prevention among older adults, has been increasing. Also, the focus of the publications has changed through the years. At the beginning of the period covered by our review, there were more publications about telehealth. In the past 4 years, publications more often report on the use of e-health and m-health tools in computer tailored lifestyle or programs that provide support and feedback for a healthy lifestyle. The results show that for different study groups, different e-health and m-health tools are used. The choice of an adequate tool depends on the specificities of the participants. This could explain why there are many different e-health tools and programs reported [15]. Another explanation for this diversity is the rapid change in the available e-health m-health tools [2]. Thus, although 40 % of the publications we reviewed, report the use of telehealth for older adults in general, this might change in the near future as new m-health tools (such as apps) are becoming available [16]. Most probably, some of these tools are already used by older adults, but are not yet studied and reported in the literature.

In this review, we also outline the evidence on the facilitating factors and barriers to the use of e-health and m-health tools for health promotion and primary prevention among older adults. The results show different facilitating factors and barriers. When the barriers are studied, over 25%of the publications mentioned the lack of motivation, support and feedback as obstacles. At the same time, the results for the facilitating factors also show that strong motivation as well as adequate support and feedback are important for the continuity of the health program based on e-health and m-health tools. It is recognized hewer that these factors are important determinants of behavior change and not necessarily direct determinants of the use of e-health and m-health tools [13]. When health promotion and primary prevention programs offer support or feedback, older adults are more likely to keep using the e-health and m-health tools offered by the program. Motivation can be stimulated in different ways [17]. The most frequently mentioned motivator is feedback on the extent to which people have achieved their goals. Such feedback can come from a professional or a peer-support group. We find evidence however, that the use of an online support group can have both positive and negative effects. If the feedback is formulated positively, it can be a motivation for further achievement. But if the group mostly focuses on the negative aspects of the use of e-health and m-health tools, and provides more negative comments, then, it can turn into a barrier. One publication indicates rewards as a facilitating factor. This could be not only a financial reward, but also the achievement of the health goals [13, 17]. To help with the motivation, adequate goal setting is an important facilitating factor [18]. When older adults have a personal aim and reachable goals, they are more likely to pursue the targeted behavior change and therefore, continue to use the e-health and m-health tools offered. If the goals are too difficult to reach then, this has negative effects, and the goal setting becomes a barrier [18, 19].

Some of the publications that we reviewed point to sociodemographical barriers. For some older adults for example, it is problematic to work with new technologies. This could be due to a low educational level or limited skills with electronic devices. When the e-health and m-health tools use technologies, which older adults already know, the ease of use is a facilitating factor. This tool should also present the information in a clear and comprehensible way. Also, the older adults’ access to health promotion or primary prevention programs, can be facilitated through telehealth [20]. With the use of this type of e-health, older adults do not have to travel to benefit from such programs [21].

Although our review was systematic and we took care to assure its quality (see Additional file 1), we still need to acknowledge some key limitations. A limitation of this review is that the search for relevant publications is done in one search engine by a single researcher. Although PubMed is the most relevant search engine with regard to our topic and it includes an enormous volume of publications, we may have missed publications on commercial e-health or m-health tools. Also, a certain bias in selecting relevant publications is present since only one researcher did the selection. Another limitation is that we assessed the study designs in a qualitative manner without applying a standardized protocol that could have helped us to quantify the strengths and weaknesses of the study designs. Therefore, our review should be only seen as a first attempt to bring together evidence on the use of e-health and m-health tools for health promotion and primary prevention among older adults.

With regard to the scope of our review, we only address the use of m-health and e-health tools for primary prevention while the use of these tools is equally relevant in secondary and tertiary prevention and in treatment. Such applications of m-health and e-health tools are widely reported in the literature [22, 23]. Also, our review is exclusively focused on older adults while a valuable starting point in future reviews could be the inclusion of more population groups or a comparison with the general population [24–26]. In addition, as stated at the outset of this paper, our review should be seen as an initial step that explores the scope of the use of e-health and m-health tools for health promotion and primary prevention among older adults. We were unable to explore the effectiveness of e-health and m-health tools among older adults. Specifically, our review captures studies with very different objectives. For example, some of the studies focus on the effectiveness of e-health and m-health programs, while others not. Even if we only examine studies that specifically focus on the effectiveness of e-health and m-health programs, the focus is not necessarily on the effectiveness of the e-health and m-health tools but rather on the effectiveness of the programs in general. Thus, the outcomes measured do not reflect the effects of e-health and m-health tools, but the more general objective of the study. Subsequent more specific reviews focused on the effectiveness of e-health and m-health tools and programs for older adults within specific settings need to be conducted to obtain a better understanding of how such programs should be designed and implemented.

## Conclusions

The results of this systematic literature review show that the relevance of e-health and m-health tools in health promotion and primary prevention among older adults is recognized and that there are a variety of uses of such tools. Also, the research focused on this issue is increasing since more publications have appeared in recent years. It seems however, that the use of e-health and m-health tools in health promotion programs for older adults, is mostly an isolated initiative, especially outside the US. European countries for example, that experience a fast population aging, could specifically benefit from the use of e-health and m-health tools in health promotion and primary prevention programs among older adults. If these programs are designed with caution to avoid potential barriers (as those outlined here), and if the cost-effectiveness of the programs can be demonstrated in future studies, governments might be willing to consider their expansion and funding. In this regard, more evidence on the effectiveness and cost-effectiveness of e-health and m-health health promotion programs for older adults is needed.

## Additional files

Additional file 1: PRISMA 2009 Checklist. (PDF 135 kb) Additional file 2: Description of the articles reviewed. (PDF 263 kb)
