# Supplementary material for: The use of e-health and m-health tools in health promotion and primary prevention among older adults: a systematic literature review
Source: BMC Health Serv Res. 2016 Sep 5;16(Suppl 5):290. doi: 10.1186/s12913-016-1522-3 (PMC5016733; doi:10.1186/s12913-016-1522-3)
Supplement: Additional file 2: — Description of the articles reviewed. (PDF 263 kb) [file 12913_2016_1522_MOESM2_ESM.pdf]

Additional File 2

Number	Title	Authors	Year of publication	Characteristics of the studies	Characteristics of e-health and m-health tools, and application	Facilitating factors for the use of e-health and m-health tools in health promotion targeting older adults	Barriers to the use of e-health and m-health tools in health promotion targeting older adults
1.	User preferences and usability of iVitality: optimizing an innovative online research platform for home-based health monitoring.	Mara van Osch,<br>AJM Rövekamp,<br>Stephanie N Bergman-Agteres,<br>Liselotte W Wijsman,<br>Sharon J Ooms,<br>Simon P Mooijaart,<br>Joan Vermeulen	2015	**Aim of study:** explorative<br><br>**Research approach:** mixed (primary data)<br><br>**Design:** qualitative<br><br>**Data collection:** observations; unstructured/ semi structured interviews	**Description of e-health:** smartphone application collects data and provides feedback<br><br>**Study group:** with risk/signs of specific disease<br><br>**Country:** Netherlands	The smartphone application was evaluated as easy to use, user-friendly, and useful. These findings are important, and suggest potential acceptance and usage of iVitality in the 6-month POP study that is currently being conducted in 150 persons who are offspring of patients with dementia.<br>Participants reported having autonomous (ie, intrinsic) motivation rather than controlled motivation regarding the use of the iVitality smartphone application. Higher autonomous motivation has been found to be associated with positive outcomes, as motivation evolves from one's personal values and is considered as part of the larger self, while controlled motivation is often linked with less beneficial outcomes.<br>Important requirements that determined usefulness and ease of use in our study were the frequency and time interval of measurements, the (amount of) questions, and the visual features of iVitality<br>Motivation is key to adherence with (home-based) research or self-monitoring protocols.	Neither participant foresaw problems in using the iVitality smartphone application<br>Participants experienced problems related to, for example, unclear notifications, menu navigations, and unclear graphical presentations. Interpretation of the proposed graphical presentation presented in Figure 2B (ie, reference lines stating mild or severe hypertension, displayed by the gray and black line, respectively) without additional explanation about what the graphs represented was difficult for participants
2.	What do general practitioners think about an online self-regulation programme for health promotion? Focus group interviews.	Jolien Plaete,<br>Geert Crombez,<br>Ann DeSmet,<br>Myriam Deveugele,<br>Maïté Verloigne,<br>Ilse De Bourdeaudhuij	2015	**Aim of study:** Explanatory<br><br>**Research approach:** Qualitative (primary data)<br>**Design:** Qualitative<br><br>**Data collection:** Focus group discussion	**Description of e-health:** Computer tailored feedback in the waiting room of general practitioners on a tablet. When patients come into the practice they are informed by their GP or by the practice assistant about the eHealth program. With feedback from the GP<br><br>**Study group:** No specific requirement<br><br>**Country:** Belgium	GPs considered the use of the principles of self-regulation (goal setting and action planning) for health promotion as a good method. Two different ways of delivery were presented: (1) using a tablet in the waiting room or,<br>(2) using a tablet during consultation., and accessible with the internet at home.	GPs mentioned that the eHealth program would not be suitable for all patients. They believe that some patients, especially elderly patients and patients with a low educational level, cannot work with a tablet or do not have an email address or a computer at home.<br>For patients with a lower educational level, the cost of mobile phones, tablets and internet connection especially leads to inequalities in accessing to these instruments<br>Barrier lack of time: An example regarding the barrier lack of time, is that the program can be halted and resumed at any time. This means that patients can choose when to finalize the different parts of the intervention<br>Another barrier might be access to the application.<br><br>Barriers Solutions<br>Lack of time • Let patients use the eHealth program on a tablet during the waiting time before<br>consultation. • Let patients start the eHealth program in practice. When time is up let them halt the<br>program and motivate them to resume it back at home.• Give an additional flyer to patients to motivate them to resume or start the eHealth program at home. Risk of theft of the tablet when used in the waiting room • Use a security system in the waiting room. Playing games on the tablet in the waiting room instead of using the eHealth program. • Use an application blocker on the tablet. Not clear where the tablet is meant for . • Use attractive posters and flyers that explain what the tablet is aiming for • For group practices: let the practice assistants explain the eHealth program to patients and let them motivate and assist patients to use the tablet. Working with an appointment system, implicating there<br>is no waiting time before consultation. • Give the tablet after the consultation and let patients use it in the waiting room.<br>In case patients cannot stay in practice, give an additional flyer with the web link on to motivate them to start the intervention at home.
3.	Can an evidence-based book club intervention delivered via a tablet computer improve physical activity in middle-aged women?	Diane K. Ehlers,<br>Jennifer L. Huberty,<br>Gert-Jan de Vreede	2015	**Aim of study:** Explanatory<br><br>**Research approach:** Quantitative (primary data)<br><br>**Design:** Quantitative - randomized controlled trial<br><br>**Data collection:** Unstructured / semi structured interviews	**Description of e-health:** The Fit Minded Workbook was designed as a supplementary tool to help women improve their PA knowledge/skills, self-efficacy, self-regulation, social support, and self-worth.<br><br>**Study group:** For woman only<br><br>**Country:** United States	Women in both groups cited the books as specifically useful for helping them adopt PA. Women in the Standard group also often cited the support they received from other group members as a key factor to helping them change their PA behaviors,	Women in the Tablet group felt that participating via videoconferencing limited their ability to fully connect with the other women.<br>Limitations included audio delays that resulted in women talking over each other, time spent resolving technical difficulties, varying levels of digital literacy within the group, and background noise/feedback. The Tablet group<br>facilitator also noted that some women spent time on other tasks during the meetings, such as making dinner, conversing with family members, answering the phone, or watching television.<br>Reasons for not reading the blogs included lack of time and interest and needing to log into the Fit Minded Web site.<br>Women reported that they found the accelerometers inconvenient to wear and often cited forgetting, occasions<br>requiring a certain type of dress (e.g., work-related, weddings), and vacationing as challenges to wearing the device

Number	Title	Authors	Year of publication	Characteristics of the studies	Characteristics of e-health and m-health tools, and application	Facilitating factors for the use of e-health and m-health tools in health promotion targeting older adults	Barriers to the use of e-health and m-health tools in health promotion targeting older adults
4.	A mixed-methods randomized controlled trial of financial incentives and peer networks to promote walking among older adults.	Jeffrey T. Kullgren, Kristin A. Harkins, Scarlett, L.Bellamy, Amy Gonzales, Yuanyuan Tao, Jingsan Zhu, Kevin G. Volpp, David A. Asch, Michele Heisler, Jason Karlawish	2014	**Aim of study:** Explanatory<br><br>**Research approach:** Mixed (primary data)<br><br>**Design:** Quantitative - randomized controlled trial<br><br>**Data collection:** Online web based questionnaires/assess ments	**Description of e-health:** pedometer intervention each day and connected their pedometer to their computer each night, with weekly automated feedback in an email or text<br><br>**Study group:** No specific requirement<br><br>**Country:** United States	Financial incentives, feedback with email or text, and the peer group should help motivate the participants.	Similarly, there are several potential reasons why the peer network intervention did not increase walking. First, nearly half of participants did not post any online messages. This suggests that for a significant number of individuals, message boards may not be successful ways to encourage communication. Next, although a subset of participants used the message board to share and solicit emotional and esteem support from their peers, many individuals used the space to share frustrations with their inability to meet walking goals.
5.	Improving access to health information for older migrants by using grounded theory and social network analysis to understand their information behaviour and digital technology use.	K.T. Goodall, L.A. Newman P.R. Ward	2014	**Aim of study:** explorative<br><br>**Research approach:** Qualitative (primary data)<br><br>**Design:** Qualitative<br><br>**Data collection:** Unstructured / semi structured interviews	**Description of e-health:** no specific application, a view on how migrants access online health information<br><br>**Study group:** Cultural group<br><br>About immigrants<br><br>**Country: Australia**	This included identifying various English language electronic and print media as important information sources which they used often instead of, or in addition to, birth language sources. These included local free-to-air television services, radio programs and a local newspaper.	Despite reports of marked increases in use by older age groups (Australian Bureau of Statistics 2011a), age continues to be a barrier to using ICTs (Heart & Kalderon2013). The older CALD migrants in our study faced a range of barriers to using various sources of information, particularly lack of English language skills for speaking, understanding or reading. It is highly likely that this is linked to their low levels of education and low levels of literacy in their birth language, which are common in this cohort. Other studies suggest that a major barrier for non-English speakers is the significant proportion of the Internet's content usually being in English (Greenstock et al. 2012).
6.	The effectiveness of loyalty rewards to promote the use of an Internet-based heart health program.	Sam Liu, Corinne Hodgson, Ahmad M Zbib, Ada YM Payne, Robert P Nolan	2014	**Aim of study:** Descriptive<br><br>**Research approach:** Desk research (secondary data)<br><br>**Design:** Qualitative - not randomized controlled trial<br><br>**Data collection:** Test results / self-report	**Description of e-health:** My Health eSupport program was a free, self-guided, fully automated healthy lifestyle program that proactively delivered tailored email messages at 2-week intervals<br><br>**Study group:** Physical conditions<br><br>**Country:** Canada	The main finding of this study was that a single exposure of loyalty rewards significantly influenced enrollment for the My Health eSupport program. Individuals were 27.9 times more likely to enroll when presented with loyalty rewards	
7.	Assessing the feasibility of using uniaxial accelerometers with an online support platform in the delivery of a community-based exercise referral scheme.	Jemma L. Hawkins, Emily J. Oliver, Jeannie Wyatt-Williams, Elaine Scale, Hugo C. van Woerden	2015	**Aim of study:** Explanatory<br><br>**Research approach:** Qualitative (primary data)<br><br>**Design:** Qualitative<br><br>**Data collection:** Unstructured / semi structured interviews	**Description of e-health:** Testing of accelerometers.<br><br>**Study group:** No specific requirement<br><br>**Country:** Wales	Those who were using the tool were positive regarding its perceived utility.	These were the following: lack of technology literacy or access, condition severity (ie, that they felt their movement was so restricted the devices would be inappropriate), or fear of costs associated with losing the device. Participants reported a lack of awareness of the scope of the system capabilities, and uncertainty regarding its operation. Crucially, the system element most aligned with contemporary theories of motivation (the online portal) was not used in the way that it was designed, with compatibility between the devices and privately owned computers a common problem. The majority of the sample did not have personal access to technology at a sufficient specification to successfully use the software.
8.	Telehealth interventio ns for primary prevention of cardiovascular disease: a systematic review and meta-analysis.	Samuel William David Merriel, Verity Andrews, Christopher Salisbury	2014	**Aim of study:** descriptive<br><br>**Research approach:** Desk research (secondary data)<br><br>**Design:** Systematic review	**Description of e-health:** Overview of different telehealth innovation for CVD risk<br><br>**Study group:** Based on setting<br><br>**Country:** United States		

Number	Title	Authors	Year of publication	Characteristics of the studies	Characteristics of e-health and m-health tools, and application	Facilitating factors for the use of e-health and m-health tools in health promotion targeting older adults	Barriers to the use of e-health and m-health tools in health promotion targeting older adults
				**Data collection:** Secondary data / patient records			
9.	A randomized comparative effectiveness study of Healthy Directions 2-- a multiple risk behavior intervention for primary care.	KarenM. Emmons, Elaine Puleo, Mary L.Greaney, MatthewW.Gillman GaryG. Bennett, JessHaines, Kim Sprunck-Harrild, K. Viswanath	2014	**Aim of study:** Explanatory<br><br>**Research approach:** Quantitative (primary data)<br><br>**Design:** Quantitative - randomized controlled trial<br><br>**Data collection:** Secondary data / patient records	**Description of e-health:** HD2 : multiple component intervention, the use of motivation, pedometer and information, and vitamins. The intervention focused on influences at the individual, interpersonal, and community levels that could motivate and maintain behavior change and be sustainable with tailored feedback rapport.<br><br>**Study group:** No specific requirement<br><br>**Country:** United States	One factor in HD2 that may have enhanced its success was allowing participants to select whether they received the intervention via print materials or a website, which may enhance engagement and satisfaction. In addition, the intervention addressed all five risk behaviors, which may have facilitated choice and engagement, as well as increasing applicability to a broad population.	
10.	Are we sure that Mobile Health is really mobile? An examination of mobile device use during two remotely-delivered weight loss interventions.	Gabrielle M. Turner-McGrievya,, Deborah F. Tate	2014	**Aim of study:** Explanatory<br><br>**Research approach:** Quantitative (primary data)<br><br>**Design:** Quantitative - randomized controlled trial<br><br>**Data collection:** Online web based questionnaires/assessments	**Description of e-health:** Weight reduction with Pounds OFF Digitally, Or MPOD which was with an app. For self-monitoring and delivered via Twitter<br><br>**Study group:** For woman only;<br><br>Physical conditions<br><br>**Country:** United States		
11.	Effects of a walking intervention using mobile technology and interactive voice response on serum adipokines among postmenopausal women at increased breast cancer risk.	Adana A. M. Llanos, Jessica L. Krok, Juan Peng, Michael L. Pennell, Mara Z. Vitolins, Cecilia R. Degraffinreid, Electra D. Paskett	2014	**Aim of study:** Explanatory<br><br>**Research approach:** Quantitative (primary data)<br><br>**Design:** Quantitative - randomized controlled trial<br><br>**Data collection:** Standardized questionnaires/interviews/surveys; Test results / self-report	**Description of e-health:** 12 week walking intervention. With interactive voice response and personal coaching<br><br>**Study group:** Physical conditions<br><br>**Country:** United States		
12.	The use of mHealth to deliver tailored messages reduces reported energy and fat intake.	Erica J. Ambeba, Lei Ye, Susan M. Sereika, Mindi A. Styn, Sushama D. Acharya, Mary Ann Sevick, Linda J. Ewing, Molly B. Conroy, Karen Glanz, Yaguang Zheng, Rachel W. Goode,	2014	**Aim of study:** Explanatory<br><br>**Research approach:** Desk research (secondary data)<br><br>**Design:** Quantitative - randomized controlled trial	**Description of e-health:** Standard behavioral intervention: (1) group sessions, (2) dietary and exercise goals, and (3) self-monitoring with the use of a PDA and with or without detailed feedback.<br><br>**Study group:** Physical conditions<br><br>**Country:** United States	The findings revealed that individuals who received a daily tailored feedback message reported reducing their energy and saturated fat intake to a greater degree than did those who did not receive the messages. The current investigation demonstrated that tailored feedback messages delivered daily in real time and in response to diary entries resulted in participants making significant improvements in dietary intake.	Compared with the 6-month intake, the increase in energy and total fat consumed that was observed at 12 months may have occurred because the participants had become desensitized to the messages, which had not been changed since the first month.

Number	Title	Authors	Year of publication	Characteristics of the studies	Characteristics of e-health and m-health tools, and application	Facilitating factors for the use of e-health and m-health tools in health promotion targeting older adults	Barriers to the use of e-health and m-health tools in health promotion targeting older adults
		Meghan Mattos,<br>Lora E. Burke		**Data collection:** Secondary data / patient records			
13.	The effectiveness of telemedicine for weight management in the MOVE! Program.	April D. Ahrendt,<br>Kendra K. Kattelmann,<br>Thomas S. Rector,<br>David A. Maddox	2014	**Aim of study:** Explanatory<br><br>**Research approach:** Desk research (secondary data)<br><br>**Design:** Qualitative - not randomized controlled trial<br><br>**Data collection:** Secondary data / patient records	**Description of e-health:** Weight Management Program.3 The MOVE! Weight Management Veterans in the treatment group attended a group weekly class series for 12 weeks (size limit 8 participants due to conference room size). These classes (1 hour each) incorporated an interdisciplinary approach to weight management and included information on diet, physical activity, and behavioral modifications<br><br>**Study group:** Physical conditions<br><br>**Country:** United States	The use of videoconferencing for people living in rural areas should be considered due to multiple barriers such as reduced access to reliable weight management programs and increased rates of obesity, heart disease, and diabetes compared to people living in urban areas. This study demonstrates that videoconferencing is an effective form of treatment for weight management.	
14.	Effects of a Web-based tailored intervention to reduce alcohol consumption in adults: randomized controlled trial.	Daniela N Schulz,<br>Math JJM Candel,<br>Stef PJ Kremers,<br>Dominique A Reinwand,<br>Astrid Jander,<br>Hein de Vries	2013	**Aim of study:** Explanatory<br><br>**Research approach:** Quantitative (primary data)<br><br>**Design:** Quantitative - randomized controlled trial<br><br>**Data collection:** Standardized questionnaires/interviews/surveys	**Description of e-health:** Alcohol-Everything Within the Limits Web-based, 3-session, tailored program targeting adult problem drinkers The personalized advice, which was presented immediately on the respondent's computer screen, consisted of 5 parts, each focusing on a different psychosocial construct of the model (i.e. knowledge, awareness, attitude, social influence, self-efficacy, and action planning).<br><br>**Study group:** Behaviors-lifestyle<br><br>**Country:** Germany		
15.	Employing virtual advisors in preventive care for underserved communities: results from the COMPASS study.	Abby C. King,<br>Timothy W. Bickmore,<br>Maria Ines Campero,<br>Leslie A. Pruitt,<br>James Langxuan Yin	2013	**Aim of study:** Explanatory<br><br>**Research approach:** Quantitative (primary data)<br><br>**Design:** Quantitative - randomized controlled trial<br><br>**Data collection:** Test results / self-report	**Description of e-health:** Pedometer at the waist on a daily basis and to download it on the virtual advisor computer via USB port each time they accessed the virtual advisor (participants did not have to manually record their steps; they simply plugged the pedometer into the computer's USB port for automatic download to the system). The virtual advisor program used the pedometer information to provide tailored feedback and advice to each intervention participant throughout the program. The senior center virtual advisor program was housed on a dedicated computer located in a small partitioned cubicle at the community center.<br><br>**Study group:** Cultural group<br><br>For Latino's<br><br>**Country:** United States		Few e-health programs have been designed for persons with lower levels of education (Viswanath & Kreuter, 2007), and few e-health programs incorporate cultural factors in their health communications (Kreuter & McClure, 2004). Such barriers, including reduced quality of health information websites that have been developed for non-English speakers (Cardelle & Rodriguez, 2005), problems consisting mostly of forgetting login information or experiencing<br>problems in trying to print educational tips.
16.	Designing and implementing a comparative effectiveness study of two strategies for delivering high quality CHD prevention: methods and participant	Stacey L. Sheridan,<br>Lindy B. Draeger,<br>Michael P. Pignone,<br>Philip D. Sloane,<br>Carmen Samuel-Hodge,<br>Eric A. Finkelstein,<br>Ziya Gizlice,<br>Maihan B. Vu,<br>Daniel P. Gitterman,<br>Shrikant I. Bangdiwala,	2013	**Aim of study:** Explanatory<br><br>**Research approach:** Quantitative (primary data)<br><br>**Design:** Quantitative - randomized controlled trial	**Description of e-health:** a combined lifestyle and medication adherence intervention delivered in two alternate formats: counselor-delivered or web-based<br><br>**Study group:** No specific requirement<br><br>**Country:** United States		

Number	Title	Authors	Year of publication	Characteristics of the studies	Characteristics of e-health and m-health tools, and application	Facilitating factors for the use of e-health and m-health tools in health promotion targeting older adults	Barriers to the use of e-health and m-health tools in health promotion targeting older adults
	characteristics for the Heart to Health study.	Katrina E. Donahue, Kelly Evenson, Alice S. Ammerman, Thomas C. Keyserlin		**Data collection:** Focus group discussion			
17.	Web-based intervention to promote physical activity by sedentary older adults: randomized controlled trial.	A. Blair Irvine, Vicky A Gelatt, John R Seeley, Pamela Macfarlane, Jeff M Gau	2013	**Aim of study:** Explanatory<br><br>**Research approach:** Quantitative (primary data)<br><br>**Design:** Quantitative - randomized controlled trial<br><br>**Data collection:** Online web based questionnaires/assessments	**Description of e-health:** Active After 55, was a multiple-visit Internet program to enhance functional ability, mobility, and physical activity of older adults. Computerized decision aid and then either 7 sessions of counseling from a counselor or 7 sessions of interactive tailored messaging on the web.<br><br>**Study group:** No specific requirement<br><br>**Country:** United States	the program was very easy to use , the overall information was very helpful, the articles provided by the program were very helpful	The 13 potential barriers included lack of willpower, no one to exercise with, fear of injury, lack of skills, lack of time, bad weather, no safe place, lack of social support, finances, being out of town, too old and out of shape, dislike of sweat, and exercise is boring.
18.	A telephone-supported cardiovascular lifestyle programme (CLIP) for lipid reduction and weight loss in general practice patients: a randomised controlled pilot trial.	Keren Louise Stuart, Belinda Wyld, Kathryn Bastiaans, Nigel Stocks, Grant Brinkworth, Phil Mohr, Manny Noakes	2012	**Aim of study:** Explanatory<br><br>**Research approach:** Quantitative (primary data)<br><br>**Design:** Quantitative - randomized controlled trial<br><br>**Data collection:** Standardized questionnaires/interviews/surveys	**Description of e-health:** CLIP group received the CLIP handbook, containing a summary dietary guide, sample menu plans and recipes based on the clinical cardiovascular dietary intervention Week 4, intervention participants received a full copy of the CSIRO Healthy Heart Program (28) commercial book publication. This coincided with the introduction of the CLIP aerobic exercise. With calls from coaches<br><br>**Study group:** Physical conditions<br><br>**Country: Australia**	The CLIP was effective in enhancing self-motivation to adopt a healthy lifestyle. This is in line with an evaluation of the TSRQ, which found that autonomous motivation was positively associated with healthier lifestyle choices such as higher levels of fruit and vegetable consumption and increased levels of physical activity.	
19.	Effectiveness of a quality improvement intervention targeting cardiovascular risk factors: are patients responsive to information and encouragement by mail or post?	Ellie Senesael, Liesbeth Borgermans, Erwin Van De Vijver, Dirk Devroey	2013	**Aim of study:** Explanatory<br><br>**Research approach:** Quantitative (primary data)<br><br>**Design:** Quantitative - randomized controlled trial<br><br>**Data collection:** Test results / self-report	**Description of e-health:** ACG received information, encouragement, and personal tips sent by post or email every 2 weeks for a period of 6 months<br><br>**Study group:** risk/signs of specific disease<br><br>**Country:** Belgium		
20.	Exercise advice by humans versus computers: maintenance effects at 18 months.	Abby C. King, Eric B. Hekler, Cynthia M. Castro, Bess H. Marcus, Robert H. Friedman, Melissa A. Napolitano	2014	**Aim of study:** Explanatory<br><br>**Research approach:** Quantitative (primary data)<br><br>**Design:** Quantitative - randomized controlled trial	**Description of e-health:** 12-month home-based moderate-intensity physical activity (primarily walking) adoption program delivered via a trained telephone counselor a similar program delivered via an automated, computer-controlled interactive telephone system or a general health education control arm<br><br>**Study group:** No specific requirement<br><br>**Country:** United States		

Number	Title	Authors	Year of publication	Characteristics of the studies	Characteristics of e-health and m-health tools, and application	Facilitating factors for the use of e-health and m-health tools in health promotion targeting older adults	Barriers to the use of e-health and m-health tools in health promotion targeting older adults
				**Data collection:** Standardized questionnaires/interviews/surveys			
21.	Effects of remote feedback in home-based physical activity interventions for older adults: a systematic review.	Hilde Geraedts, Agnes Zijlstra, Sjoerd K. Bulstra, Martin Stevens, Wiebren Zijlstra	2012	**Aim of study:** descriptive<br><br>**Research approach:** Desk research (secondary data)<br><br>**Design:** Systematic review<br><br>**Data collection:** Secondary data / patient records	**Description of e-health:** Only about remote feedback in home based physical activity<br><br>**Study group:** No specific requirement<br><br>**Country:** The Netherlands	Positive effect of remote feedback	
22.	DASH to wellness: emphasizing self-regulation through e-healthin adults with prehypertension.	Ashley E. Dorough, Richard A. Winett, Eileen S. Anderson, Brenda M. Davy, Emily C. Martin, Valisa Hedrick	2012	**Aim of study:** Explanatory<br><br>**Research approach:** Quantitative (primary data)<br><br>**Design:** Quantitative - randomized controlled trial<br><br>**Data collection:** Test results / self-report	**Description of e-health:** Weekly, participants in D2W plus monitored and electronically reported on key lifestyle behaviors with the DASH diary and the wellness tracker. For the duration of the 10-week program they received weekly electronic two DASH 2 wellness newsletters, providing (a) support for lifestyle modification, content, and self-regulation strategies based on social– cognitive theory (Winett et al., 2011); and (b) individualized, electronic feedback based on their reported data about weight (maintain weight or a small weight loss), steps per day<br><br>**Study group:** Physical conditions<br><br>**Country:** United States		
23.	Interactive voice response for relapse prevention foll owing cognitive-behavioral therapy for alcohol use disorders: a pilot study.	Gail L. Rose, Joan M. Skelly, Gary J. Badger, Magdalena R. Naylor, John E. Helzer	2012	**Aim of study:** descriptive<br><br>**Research approach:** Qualitative (primary data)<br><br>**Design:** Qualitative; Qualitative - not randomized controlled trial<br><br>**Data collection:** Test results / self-report	**Description of e-health:** Treatment consisted of 12 weekly 90-minute Group CBT sessions. Interactive Voice Response (ATIVR), was delivered via an automated computer-driven telephone system. There are five components to the ATIVR: 1 daily questionnaire ;2 therapeutic questionnaire; 3 didactic skills review; 4 coping skills practice; 5 therapist feedback messages<br><br>**Study group:** Behaviors-lifestyle<br><br>**Country:** United States		Participants who did not call typically said it was because they forgot, although some reported they did not need the ATIVR because they were doing well and/or had other supports in place.
24.	Multiple behavior changes in diet and activity: a randomized controlled trial using mobile technology.	Bonnie Spring, Kristin Schneider, H. Gene McFadden, Jocelyn Vaughn, Andrea T. Kozak, Malaina Smith, Arlen C. Moller, Leonard H. Epstein, Andrew DeMott, Donald Hedeker, Juned Siddique, Donald M. Lloyd-Jones	2012	**Aim of study:** Explanatory<br><br>**Research approach:** Quantitative (primary data)<br><br>**Design:** Quantitative - randomized controlled trial<br><br>**Data collection:** Test results / self-report	**Description of e-health:** During the 2-week baseline phase, participants wore an accelerometer, recorded diet and activity on the handheld device, and uploaded data daily. Coaches tailored behavioral strategies based on participants' baseline data. Participants used a personal digital assistant to record and self-regulate their targeted behaviors. They were advised to carry the device and record immediately after executing a behavior. Participants were expected to reach their behavioral targets during treatment week 2 and to maintain them during week 3. During the 3 treatment weeks, participant's uploaded data daily and communicated as needed with their coaches via telephone or e-mail, per preference, to overcome challenges.<br><br>**Study group:** Behaviors-lifestyle		Non adherence with lifestyle change advice is cited as a major barrier to effective preventive care. Many physicians express skepticism that patients will change unhealthy behaviors; they also report lack of time and training to counsel patients effectively.

Number	Title	Authors	Year of publication	Characteristics of the studies	Characteristics of e-health and m-health tools, and application	Facilitating factors for the use of e-health and m-health tools in health promotion targeting older adults	Barriers to the use of e-health and m-health tools in health promotion targeting older adults
					**Country:** United States		
25.	Obesity treatment for socioeconomically disadvantaged patients in primary care practice.	Gary G. Bennett, Erica T. Warner, Russell E. Glasgow, Sandy Askew, Julie Goldman, Debra P. Ritzwoller, Karen M. Emmons, Bernard A. Rosner, Graham A. Colditz	2012	**Aim of study:** Explanatory<br><br>**Research approach:** Quantitative (primary data)<br><br>**Design:** Quantitative - randomized controlled trial<br><br>**Data collection:** Online web based questionnaires/assess ments	**Description of e-health:** Patients are prescribed 3 tailored goals to modify routine obesogenic lifestyle behaviors Behavior change goals were modeled that were tailored to the patient New goals were selected at subsequent 13-week intervals. For the duration of the study, participants maintained a hypertension medication adherence goal (to take their medication as prescribed daily). Participants chose to self-monitor their progress using either the study website or an interactive voice response system, available in English and Spanish. Both tracking systems provided real-time tailored feedback. Trained community health educators delivered counseling calls monthly during the first 12 months of intervention and bimonthly during the second year (18 total scheduled calls).<br><br>**Study group:** Physical conditions<br><br>**Country:** United States		
26.	Usability and feasibility of mobile phone diaries in an experimental physical exercise study.	Reetta Heinonen, Riitta Luoto, Pirjo Lindfors, Clas-Ha°kan Nyga	2012	**Aim of study:** Explanatory<br><br>**Research approach:** Quantitative (primary data)<br><br>**Design:** Quantitative - randomized controlled trial<br><br>**Data collection:** Online web based questionnaires/assess ments	**Description of e-health:** The exercise program included aerobic training four times a week, 50 min per session, with a progressive increase in intensity. Adherence to the trial was supported by an option to participate in supervised aerobics or step aerobics sessions at the research institute twice a week. With the use of an mobile phone diary to collect data on symptoms and amount of physical exercise<br><br>**Study group:** For woman only<br><br>**Country:** Finland	The mobile phone diary was considered a fast and easy way for participants to respond to questionnaires. Users commented that doing the diary "has become a routine." It was also noted that "since mobile phones are part of everyday life, it is easy to remember to fill in the questionnaires."	‘‘Which factors did you find negative when using the mobile phone diary?’’ The most common answer was "nothing." Negative comments included technical problems such as network connection problems.
27.	Exploratory study of web-based planning and mobile text reminders in an overweight population.	Anastasia Soureti, Peter Murray, Mark Cobain, Mai Chinapaw, Willem van Mechelen, Robert Hurling	2011	**Aim of study:** explorative<br><br>**Research approach:** Quantitative (primary data)<br><br>**Design:** Quantitative - randomized controlled trial<br><br>**Data collection:** Online web based questionnaires/assess ments	**Description of e-health:** Participants who received the planning tool selected from a list of 13 situations, in which they were tempted to eat unhealthily and then chose an approach to change their behavior from a list of 13 solutions. Motivational cues were divided into 3 main situations: (1) experiencing positive affect, (2) experiencing negative affect, and (3) being faced with cravings After completing the planning session, participants in the PTT entered their mobile number and chose a time band to receive text reminders of their plans.<br><br>**Study group:** Physical conditions<br><br>**Country: United Kingdom**		
28.	Can eHealth tools enable health organizations to reach their target audience?	Lawrence J. Appel, Jeanne M. Clark, Hsin-Chieh Yeh, Nae-Yuh Wang, Janelle W. Coughlin, Gail Daumit, Edgar R. Miller III, Arlene Dalcin, Gerald J. Jerome,	2014	**Aim of study:** Explanatory<br><br>**Research approach:** Quantitative (primary data)<br><br>**Design:** Quantitative - randomized controlled trial	**Description of e-health:** Coaches Delivered the interventions in collaboration with the PCP to promote weight loss Focused on key weight-management behaviors (reduced calorie intake as part of the DASH diet, increased exercise, regular log-in to the study Web site, and use of food records)<br>Used motivational interviewing techniques (e.g., asking open-ended questions, exploring participants' feelings of ambivalence, supporting their optimism regarding change, and directing conversations toward the desired behavioral goals) Received case-management support	The effectiveness of remote support is particularly noteworthy because of the flexibility it offers to both participants and coaches and because it is scalable.	implementing programs similar to those used in the intervention groups in primary care could help stem the tide of obesity-related disease, but it would also require changes in healthcare delivery systems and reimbursement policies

Number	Title	Authors	Year of publication	Characteristics of the studies	Characteristics of e-health and m-health tools, and application	Facilitating factors for the use of e-health and m-health tools in health promotion targeting older adults	Barriers to the use of e-health and m-health tools in health promotion targeting older adults
		Steven Geller,<br>Gary Noronha,<br>Thomas Pozefsky,<br>Jeanne Charleston,<br>Jeffrey B. Reynolds,<br>Nowella Durkin,<br>Richard R. Rubin,<br>Thomas A. Louis,<br>Frederick L. Brancati		**Data collection:** Online web based questionnaires/assess ments	Web-based support Provided learning modules consisting of objectives, educational content, quizzes, and worksheets Provided self-monitoring tools and graphs (to record weight, minutes of exercise per day, and calories consumed per day), with a recommendation to record weight at least weekly on the study Web site Provided feedback regarding weight-loss progress (e.g., change in weight since last log-in and weight trend.<br><br>**Study group:** Physical conditions<br><br>**Country:** United States		
29.	Comparative effectiveness of weight-loss interventions in clinical practice.	Lawrence J. Appel,<br>Jeanne M. Clark,<br>Hsin-Chieh Yeh,<br>Nae-Yuh Wang,<br>Janelle W. Coughlin,<br>Gail Daumit,<br>Edgar R. Miller III,<br>Arlene Dalcin,<br>Gerald J. Jerome,<br>Steven Geller,<br>Gary Noronha,<br>Thomas Pozefsky,<br>Jeanne Charleston,<br>Jeffrey B. Reynolds,<br>Nowella Durkin,<br>Richard R. Rubin,<br>Thomas A. Louis,<br>Frederick L. Brancati	2011	**Aim of study:** Explanatory<br><br>**Research approach:** Quantitative (primary data)<br><br>**Design:** Quantitative - randomized controlled trial<br><br>**Data collection:** Online web based questionnaires/assess ments	**Description of e-health:** Coaches Delivered the interventions in collaboration with the PCP to promote weight loss Focused on key weight-management behaviors (reduced calorie intake as part of the DASH diet, increased exercise, regular log-in to the study Web site, and use of food records)<br>Used motivational interviewing techniques (e.g., asking open-ended questions, exploring participants' feelings of ambivalence, supporting their optimism regarding change, and directing conversations toward the desired behavioral goals) Received case-management support<br>Web-based support Provided learning modules consisting of objectives, educational content, quizzes, and worksheets Provided self-monitoring tools and graphs (to record weight, minutes of exercise per day, and calories consumed per day), with a recommendation to record weight at least weekly on the study Web site Provided feedback regarding weight-loss progress (e.g., change in weight since last log-in and weight trend.<br><br>**Study group:** Physical conditions<br><br>**Country:** United States	Although in our study a disease-management company delivered the intervention restricted to remote support only, other groups, including large physician practices and insurers, could implement such programs, which could also be part of patient-centered medical home initiatives.<br><br>The effectiveness of remote support is particularly noteworthy because of the flexibility it offers to both participants and coaches and because it is scalable.	implementing programs similar to those used in the intervention groups in primary care could help stem the tide of obesity-related disease, but it would also require changes in healthcare delivery systems and reimbursement policies
30.	Telehealth stroke education for rural elderly Virginians.	Patricia A. Schweickert,<br>Carolyn M. Rutledge,<br>David C. Cattell-Gordon,<br>Nina J. Solenski,<br>Mary E. Jensen,<br>Sheila Branson,<br>John R. Gaughen	2011	**Aim of study:** Explanatory<br><br>**Research approach:** Mixed (primary data)<br><br>**Design:** Qualitative - not randomized controlled trial<br><br>**Data collection:** Standardized questionnaires/intervie ws/surveys	**Description of e-health:** The researcher presented a 20-min stroke prevention education session based on National Institute of Neurologic Disorders and Stroke-National Institute of Health (NINDS-NIH) stroke information. The education session began with an 8-min video and was followed by a discussion of stroke risk factors and prevention using Power-Point slides. The content included stroke definitions, symptoms, risk factors, risk reduction, and actions to take if someone has a stroke or transient ischemic attack. The session was interactive with participants asking and answering questions.<br><br>**Study group:** No specific requirement<br><br>**Country:** United States	The most important benefit of telemedicine is the improved access to care for patients who live in medically underserved areas where there are considerable barriers to care for disabilities such as in stroke. A systematic review of telehealth and diabetes care revealed it to be beneficial and useful, showing success with group education	
31.	Formative evaluation of the telecare fall prevention project for older veterans.	Isomi M Miake-Lye,<br>Angel Amulis,<br>Debra Saliba,<br>Paul G Shekelle,<br>Linda K Volkman,<br>David A Ganz	2011	**Aim of study:** descriptive<br><br>**Research approach:** Qualitative (primary data)	**Description of e-health:** Fall prevention with telecare<br><br>**Study group:** No specific requirement<br><br>**Country:** United States	Five patients noted the following project advantages: making them well informed about falls, helping them feel prepared with home safety and prevention advice, and causing them to be more alert to the issue of falls. Two patients also mentioned the emotional benefit of the project, which made them feel cared about. Our experience implementing the Telecare fall prevention pilot project suggests that geographic factors are very important in determining the effectiveness of telephone consultation and triage.	Although this project feature in and of itself was emphasized as a success by patients and employees alike, patients who were found to need in-person follow-up services then faced a dilemma. limited resources, specifically the tight schedules and large caseloads of both Telecare and the VA system in general

Number	Title	Authors	Year of publication	Characteristics of the studies	Characteristics of e-health and m-health tools, and application	Facilitating factors for the use of e-health and m-health tools in health promotion targeting older adults	Barriers to the use of e-health and m-health tools in health promotion targeting older adults
				**Design:** Qualitative<br><br>**Data collection:** Unstructured / semi structured interviews			
32.	Six-month programme on lifestyle changes in primary cardiovascular prevention: a telemedicine pilot study.	Palmira Bernocchi, Doriana Baratti, Emanuela Zanelli, Silvana Rocchi, Massimo Salvetti, Anna Paini, Simonetta Scalvini	2011	**Aim of study:** Explanatory<br><br>**Research approach:** Quantitative (primary data)<br><br>**Design:** Qualitative - not randomized controlled trial<br><br>**Data collection:** Test results / self-report	**Description of e-health:** Telemedicine program, Patients were entrusted to a nurse-tutor and followed a program of remote monitoring at home (tele monitoring with devices, scheduled tele management appointment, and tele assistance) previously described. Physical program had three basic activities: (i) 5–10-min warming, (ii) 20-min active exercise, and (iii) 5–10-min cool-down phase.<br><br>**Study group:** No specific requirement<br><br>**Country:** Italy		
33.	Reducing cardiovascular disease risk in medically underserved urban and rural communities.	Alfred A. Bove, William P. Santamore, Carol Homko, Abul Kashem, Robert Cross, Timothy R. McConnell, Gail Shirk, Francis Menapace	2011	**Aim of study:** Explanatory<br><br>**Research approach:** Quantitative (primary data)<br><br>**Design:** Quantitative - randomized controlled trial<br><br>**Data collection:** Standardized questionnaires/interviews/surveys;<br><br>Test results / self-report	**Description of e-health:** Telemedicine versus nurse management. The application of e-health was the use of telemedicine in rural and urban communities.<br><br>**Study group:** No specific requirement<br><br>**Country:** United States	Communication in both groups between subjects, the research nurses, and the care providers created the needed incentive to improve CVD risk.	
34.	The drop it at last study: six-month results of a phone-based weight loss trial.	Nancy E. Sherwood, Robert W. Jeffery, Ericka M. Welsh, Jeff VanWormer, Ann Marie Hotop	2009	**Aim of study:** Explanatory<br><br>**Research approach:** Quantitative (primary data)<br><br>**Design:** Quantitative - randomized controlled trial<br><br>**Data collection:** Test results / self-report	**Description of e-health:** Participants were sent an instructional manual, a pedometer, and log booklets for monitoring daily weight, food intake, and physical activity. After 1 week of mailing out notification letters and materials, participants in the telephone intervention conditions were contacted by their respective telephone counselors to initiate treatment. Self-directed participants received the same instructional manual, pedometer, and log booklets, but were not contacted by DIAL staff members other than to complete follow-up measures, described below. Further phone counseling<br><br>**Study group:** Physical conditions<br><br>**Country:** United States	The findings clearly indicated that a greater number of completed phone sessions was associated with significantly greater weight loss. We suspect that this was at least partially because of stronger intervention messaging around eating and activity goals, as well as the offering of more phone sessions, in the DIAL pilot trial relative to the WTB trial.. Caused for more participant who had weight loss, in comparison with their previous study	Costs of normal behavioral weight loss programs, Although the individualized phone based approach evaluated in this study has much to offer with respect to convenience and flexibility, the lack of face-to-face and/or group interactions may have decreased the social support element of treatment and in turn dampened weight loss effects
35.	Using the internet to translate an evidence-based lifestyle intervention into practice.	Kathleen M. McTigue, Molly B. Conroy, Rachel Hess, Cindy L. Bryce, Anthony B. Fiorillo, Gary S. Fischer, N. Carole Milas, Laurey R. Simkin-Silverman	2009	**Aim of study:** Explanatory<br><br>**Research approach:** Quantitative (primary data)	**Description of e-health:** The program comprised five components accessed from a home page. Automated e-mail prompts reminded participants of pending lessons, and other tasks. Each 30–45-minute lesson was audio narrated and included an optional quiz. Sixteen weekly lessons comprised the core curriculum of the DPP Lifestyle Intervention, slightly modified for online delivery. They were followed by eight monthly lessons, adapted from relevant supplemental DPP materials.	This project demonstrates that an evidence-based intensive lifestyle intervention can be adapted to an online setting and delivered in coordination with primary care medicine. Automating the educational component of the program allowed the coaches to focus their time on supporting behavior change and tailoring dietary and physical activity advice to specific participant needs	

Number	Title	Authors	Year of publication	Characteristics of the studies	Characteristics of e-health and m-health tools, and application	Facilitating factors for the use of e-health and m-health tools in health promotion targeting older adults	Barriers to the use of e-health and m-health tools in health promotion targeting older adults
				**Design:** Qualitative - not randomized controlled trial<br><br>**Data collection:** Test results / self-report	**Study group:** risk/signs of specific disease<br><br>**Country:** United States		
36.	Beyond epidemiology: field studies and the physiology laboratory as the whole world.	Hiroshi Nose,<br>Mayuko Morikawa,<br>Toshiaki Yamazaki,<br>Ken-ichi Nemoto,<br>Kazunobu Okazaki,<br>Shizue Masuki,<br>Yoshi-ichiro Kamijo,<br>Hirokazu Gen-no	2009	**Aim of study:** Explanatory<br><br>**Research approach:** Quantitative (primary data)<br><br>**Design:** Qualitative - not randomized controlled trial<br><br>**Data collection:** Test results / self-report	**Description of e-health:** three exercise training formats for middle-aged and older people: (1) interval walking training (IWT), (2) use of a portable calorimeter, and (3) the e-Health Promotion System<br><br>**Study group:** No specific requirement<br><br>**Country:** Japan	This higher adherence to the exercise training program might have been due to the fact that it was matched to individual w ˙VO2peak and that instructions for IWT based on individual walking records were passed to individuals by trainers every 2 weeks through the internet. Accordingly, subjects were able to recognize their increased physical fitness from energy expenditure and time for fast walking as IWT advanced, which encouraged them to continue with confidence that their efforts were being rewarded.	
37.	Effects of a non-face-to-face behavioral weight-control program among Japanese overweight males: a randomized controlled trial.	Minori Tanaka,<br>Yoshiko Adachi,<br>Kyo Adachi<br>Chifumi Sato	2009	**Aim of study:** Explanatory<br><br>**Research approach:** Quantitative (primary data)<br><br>**Design:** Quantitative - randomized controlled trial<br><br>**Data collection:** Standardized questionnaires/interviews/surveys;<br><br>Test results / self-report	**Description of e-health:** KTP was a completely non-face-to-face commercial program the educational elements of KTP included a booklet on behavioral weight control, self-assessment of daily behaviors, target behavior setting, and self-monitoring of daily body weight and targeted behaviors. This process was assisted twice by computer-tailored advises based on the responses to the questionnaire.<br><br>**Study group:** Physical conditions<br><br>**Country:** Japan		
38.	E-health for older people: the use of technology in health promotion.	Mimi M. Y. Tse,<br>Kim C. Y. Choi,<br>Rincy S.W. Leung	2008	**Aim of study:** descriptive<br><br>**Research approach:** Qualitative (primary data)<br><br>**Design:** Qualitative<br><br>**Data collection:** Unstructured / semi structured interviews	**Description of e-health:** Training to introduce basic knowledge of operating a computer and accessing the internet, and to promote awareness of e-health by using a computer.<br><br>**Study group:** Low computer knowledge<br><br>**Country:** Hong Kong	With the collaboration of community services, the use of technology in the form of an e-health program would be an effective tool in providing health education to older people Continual and regular provision of ehealth programs for older people will bring positive health outcomes to older adults and to society as a whole	Physical disabilities. "Poor eye–hand coordination makes it difficult for us to use the mouse." "When sitting for a period of time, I get severe low back pain. It annoys me." Language barrier and lower education level. "We don't know many words." "People think we are old fashioned, so there's no need to learn about computers." "We can't even type. How are we supposed to manage Chinese typing?" Lack of guidance. "I don't know where to get help; my friends don't know about computers, and they cannot discuss computer use with me." "I don't know the addresses of these Web sites."
39.	Cardiovascular risk reduction via telehealth: a feasibility study.	Anne M Paus Jenssen,<br>Betty Anne Spooner,<br>Merne P Wilson,<br>Thomas W Wilson	2007	**Aim of study:** Explanatory<br><br>**Research approach:** Quantitative (primary data)<br><br>**Design:** Qualitative - not randomized controlled trial	**Description of e-health:** At the initial visit, Patients had been prebooked and had completed laboratory testing. Each patient was seen by one nurse, one physician, as well as the dietician and the fitness consultant. For each patient, goals were negotiated for weight, blood pressure, lipid profile, blood glucose and smoking cessation. The dietician went to the local food store with patients to point out healthy food choices. The exercise therapist demonstrated several aerobic, strength and balance exercises. Patients were recalled at three, six, nine and 12 months. The telehealth technician measured weight (using a digital scale), waist circumference and blood pressure. Patients,	Patients stated that it was more convenient to have healthcare provided to them in this manner, and was cost-saving compared with travelling to Saskatoon. This perhaps contributed to the better follow-up rate seen in the trial group	

Number	Title	Authors	Year of publication	Characteristics of the studies	Characteristics of e-health and m-health tools, and application	Facilitating factors for the use of e-health and m-health tools in health promotion targeting older adults	Barriers to the use of e-health and m-health tools in health promotion targeting older adults
				**Data collection:** Test results / self-report	as a group, met with the nurse, the dietician and the fitness consultant.<br><br>**Study group:** No specific requirement<br><br>**Country:** Canada		
40.	Effect of emailed messages on return use of a nutrition education website and subsequent changes in dietary behavior.	W Gill Woodall, David B Buller, Laura Saba, Donald Zimmerman, Emily Waters, Joan M Hines, Gary R Cutter, Randall Starling	2007	**Aim of study:** Explanatory<br><br>**Research approach:** Quantitative (primary data)<br><br>**Design:** Quantitative - randomized controlled trial<br><br>**Data collection:** Unstructured / semi structured interviews; Online web based questionnaires/assess ments	**Description of e-health:** The "5 a Day, the Rio Grande Way" website contained content on the health benefits of fruits and vegetables; instructions for buying, storing, and preparing fruits and vegetables; and ways to increase fruits and vegetables in the family diet, particularly with children. This website in combination with emailed messages on return. So that people would more often use the website.<br><br>**Study group:** No specific requirement<br><br>**Country:** United States		
41.	One-year follow-up of a therapeutic lifestyle intervention targeting cardiovascular disease risk.	Andrew Wister, Nadine Loewen, Holly Kennedy-Symonds, Brian McGowan, Bonnie McCoy, Joel Singer	2007	**Aim of study:** Explanatory<br><br>**Research approach:** Quantitative (primary data)<br><br>**Design:** Quantitative - randomized controlled trial<br><br>**Data collection:** Test results / self-report	**Description of e-health:** The intervention consisted of a report card (sent to the participant and his or her family doctor) showing the person's Risk profile, coupled with a Telehealth-guided self-care Comparisons with previous report card grades were discussed with the participant to set new goals. Telehealth counselling occurred within 10 days of the patient receiving the annual report card and every 6 months thereafter for approximately 30 minutes per session, up to 60 minutes per year<br><br>**Study group:** No specific requirement<br><br>**Country:** Canada		
42.	Effects of nurse-managed telemonitoring on blood pressure at 12-month follow-up among urban African Americans	Nancy T. Artinian, John M. Flack, Cheryl K. Nordstrom, Elaine M. Hockman Olivia G.M. Washington, Kai-Lin Catherine Jen; Maryam Fathy	2007	**Aim of study:** Explanatory<br><br>**Research approach:** Quantitative (primary data)<br><br>**Design:** Quantitative - randomized controlled trial<br><br>**Data collection:** Standardized questionnaires/intervie ws/surveys	**Description of e-health:** There were three components to the intervention protocol; that is, participants were to take their BPs, send their BPs, and participate in tele counseling calls with the intervention nurse<br><br>**Study group:** Cultural group<br><br>For African Americans<br><br>**Country:** United States		
43.	Reach, engagement, and retention in an Internet-based weight loss program in a multi-site randomized controlled trial.	Russell E Glasgow, Candace C Nelson, Kathleen A Kearney, Robert Reid, Debra P Ritzwoller, Victor J Strecher, Mick P Couper,	2007	**Aim of study:** Explanatory<br><br>**Research approach:** Quantitative (primary data)	**Description of e-health:** The Health Media Balance program is a 6-week online self-help program that uses data from a baseline assessment to create an individually tailored weight management plan. Dimensions on which the program is tailored include health and medical history, prior weight loss efforts, intrinsic and extrinsic motivators for managing weight, perceived barriers to change, attitudes and stereotypes about overweight	Demographic and motivational factors also predicted ongoing engagement (but not initial engagement).	Our results were mixed on this issue: although we did attract many older patients with chronic illness, the enrollment analyses suggest that in general this program did not attract those at highest risk (smokers, older adults, etc) at the same rate as those at lower risk. However, among members in HMO 1 receiving letters of invitation, patients with CAD or diabetes were substantially more likely to participate than members without known chronic illness (10% and 7%, respectively, vs 2.4%).

Number	Title	Authors	Year of publication	Characteristics of the studies	Characteristics of e-health and m-health tools, and application	Facilitating factors for the use of e-health and m-health tools in health promotion targeting older adults	Barriers to the use of e-health and m-health tools in health promotion targeting older adults
		Beverly Green, Kevin Wildenhaus		**Design:** Quantitative - randomized controlled trial<br><br>**Data collection:** Standardized questionnaires/interviews/surveys	people and weight loss, social support systems, body image, nutritional habits, and physical activity.<br><br>**Study group:** Physical conditions<br><br>**Country:** United States		
44.	Evaluation of a website-delivered computer-tailored intervention for increasing physical activity in the general population.	Heleen Spittaels, Ilse De Bourdeaudhuij, Corneel Vandelanotte	2009	**Aim of study:** Explanatory<br><br>**Research approach:** Quantitative (primary data)<br><br>**Design:** Quantitative - randomized controlled trial<br><br>**Data collection:** Online web based questionnaires/assessments	**Description of e-health:** The content of this computer-tailored program was based on a previously developed and evaluated computer program on CD-ROM The assessment questionnaire contained questions on demographics, PA and psychosocial determinants of PA and was designed to provide feedback to participants on their PA level, together with tips and suggestions to improve their behavior. The advice was tailored on stages of changes both by content and the way in which the participants were approached (a more distant way for pre-contemplators to avoid resistance, a more personal way for contemplators, a decisive way for preparatory and a supporting way for participants in action or maintenance stage)<br><br>**Study group:** No specific requirement<br><br>**Country:** Belgium		Some participants who received repeated feedback also reported to have taken profit of their second advice, which shows that some individuals need additional time or stimuli before making behavioral changes.
45.	Cardiovascular disease prevention for underserved patients using the Internet: bridging the digital divide.	Michele M. Masucci, Carol Homko, William P. Santamore, Philip Berger, Timothy R. Mcconnell, Gail Shirk, Francis Menapace, Alfred A. Bove	2006	**Aim of study:** Explanatory<br><br>**Research approach:** Quantitative (primary data)<br><br>**Design:** Qualitative - not randomized controlled trial<br><br>**Data collection:** Test results / self-report	**Description of e-health:** Each participant received a manual blood pressure monitor and was instructed on its use by a registered nurse. Participants received a logbook to record: daily blood pressure, weight, and cigarette consumption. Nurses helped the participant measure his/her blood pressure and recorded these values in the logbook for later transmission via the telemedicine system.<br>All participants received computer training. Initial computer experience was determined through a questionnaire. The participants made their first time data entry with coaching by a member of the research team. The participants were instructed to monitor their blood pressures at home on a daily basis and send data sets (blood pressure) 1 week post training.<br><br>**Study group:** risk/signs of specific disease<br><br>**Country:** United States	High (4) or very high (5) scores on all computer skills had positive effects on successful use of the telemedicine system. Ownership of a computer and education level did not appear to be an impediment to participation in this program.	Internet access issues may limit the successful application of telemedicine.<br><br>Computer training is an essential element needed to address the challenges that will be faced by underserved patients to benefit from Internet
